# Examining the association of neighborhood conditions on attention‐deficit/hyperactivity disorder symptoms in autistic youth using the child opportunity index 2.0

**DOI:** 10.1002/jcv2.12267

**Published:** 2024-07-18

**Authors:** Catrina A. Calub, Irva Hertz‐Picciotto, Deborah Bennett, Julie B. Schweitzer

**Affiliations:** ^1^ Department of Psychiatry and Behavioral Sciences University of California Sacramento California USA; ^2^ MIND Institute University of California Sacramento California USA; ^3^ Department of Public Health Sciences University of California Davis California USA

**Keywords:** ADHD, adolescence, autism, early life experience, environmental influences

## Abstract

**Background:**

While neighborhood conditions have previously been shown to have substantial effects on later occupational, educational and health outcomes, this is the first study to examine the relation between neighborhood factors and attention‐deficit/hyperactivity disorder (ADHD) symptoms in children with autism and developmental delays.

**Methods:**

Children from the CHARGE (Childhood Autism Risks from Genetics and the Environment) Study were evaluated at ages 2–5 years and then later in the ReCHARGE (follow‐up) Study at ages 8–20 years (mid‐childhood/adolescence). Using linear regression, we assessed associations between the Child Opportunity Index 2.0 (COI) at birth, a multidimensional neighborhood measure of childhood opportunity, and ADHD symptoms on the Aberrant Behavior Checklist at mid‐childhood/adolescence.

**Results:**

Participants included a total of 524 youth (401 males; 123 females), composed of 246 autistic children (AUT), 85 children with Developmental Delays (DD) without autism, and 193 Typically Developing (TD) children. Mean age was 3.8 years (*SD* = 0.79) when evaluated at CHARGE and 13.5 years (*SD* = 3.69) when evaluated at ReCHARGE. Regression analyses revealed COI at birth significantly predicted ADHD symptoms during mid‐childhood/adolescence and early childhood diagnosis modified the COI effect. More specifically, COI significantly predicted ADHD symptoms in the AUT group, but not the TD or DD groups. Additional regression analyses indicated that this interaction was only present in the Social and Economic COI domain. Secondary analyses revealed autistic youth with High and Low Social and Economic COI domain scores had similar levels of ADHD symptoms during early childhood, but by mid‐childhood/adolescence, those with low Social and Economic COI domain scores had higher ADHD symptoms.

**Conclusions:**

Among autistic, but not TD or DD youth, poorer neighborhood conditions at birth predict greater ADHD symptoms in later development. These findings have important clinical implications and highlight the need for increased and improved resources in poorer neighborhoods to reduce existing disparities in ADHD, a common neurodevelopmental impairment.

## Graphical abstract

The association between poorer neighborhood conditions at birth and greater ADHD symptoms in later development is stronger in autistic individuals relative to those with developmental delays and typically developing peers. These findings have important clinical implications and highlight the need for increased and improved resources in poorer neighborhoods to reduce existing disparities.

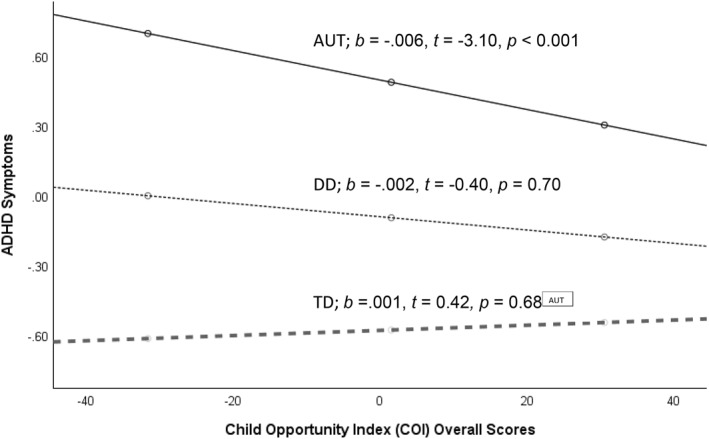

**Key points**

- Neighborhood conditions have long lasting effects on children's occupational, educational and health outcomes, yet it is currently unknown how neighborhood factors influence ADHD symptoms in children with developmental disabilities.
- Poorer neighborhood conditions at birth predict greater ADHD symptoms in later development in autistic individuals, whereas this relation is not significant in those with developmental delays without autism or in typically developing children.
- Neighborhood‐level social factors and economic resources appear particularly influential in predicting which autistic youth are at risk for ADHD symptoms in mid‐childhood/adolescence.
- Autistic youth with high and low social and economic neighborhood opportunities have similar levels of ADHD symptoms during early childhood, but by mid‐childhood/adolescence, autistic youth who, at birth, resided in neighborhoods with high social and economic opportunities have significantly fewer ADHD symptoms.
- These findings highlight the need for increased and improved resources in poorer neighborhoods to reduce existing disparities.

## INTRODUCTION

A majority (40%–70%) of autistic children exhibit clinically elevated symptoms of attention‐deficit/hyperactivity disorder (ADHD) (Lyall et al., 2017; Rommelse et al., 2010). These high rates are concerning given that ADHD symptoms are associated with greater functional impairment (Yerys et al., 2009) and poorer response to treatment (Antshel et al., 2011; Calub et al., 2022) in autistic children. A recent systematic review indicated autistic youth with ADHD experience elevated behavioral, emotional, adaptive, and cognitive difficulties compared to autistic youth without ADHD (Rosello et al., 2022). ADHD symptoms are also highly prevalent in those with developmental disabilities without autism (Baker et al., 2010) and are associated with greater conduct, language, adaptive and social deficits (Handen & Valdes, 2007). As such, it is important to understand the factors that impact ADHD symptoms among youth with neurodevelopmental conditions.

ADHD is strongly influenced by genetics (Faraone & Larsson, 2019; Tick et al., 2016); however, investigations have increasingly documented that the environment also has substantial effects on ADHD symptoms. For example, familial characteristics such as household socioeconomic status (SES), parental distress, family conflict, and parenting style are associated with ADHD symptoms (Duh‐Leong et al., 2020; Miller et al., 2018; Musser et al., 2016; Russell et al., 2016). Environmental factors outside of the family, such as the neighborhood in which a child lives, have also recently drawn interest. Neighborhood‐level SES has been shown to increase the odds of ADHD, even after controlling for family income (Razani et al., 2015; Sundquist et al., 2015; Xue et al., 2005). Other neighborhood characteristics such as less greenspace (Sakhvidi et al., 2022; Yuchi et al., 2022), fewer amenities (Reuben et al., 2020; Taylor & Kuo, 2009), presence of vandalism and dilapidated housing (Butler et al., 2012), and low perceived safety and low social support (Dahal et al., 2018; Derauf et al., 2015; Duh‐Leong et al., 2020; Razani et al., 2015) increase the risk for childhood ADHD. Collectively, it is well‐documented that neighborhood characteristics are associated with ADHD; however, to our knowledge, no study to date has yet examined the relation between neighborhood factors and ADHD symptoms in either autistic children or those with developmental delays.

The Child Opportunity Index 2.0 (COI) provides a multidimensional metric for examining the effect of the neighborhood on youth. The COI provides information on neighborhoods across three domains‐ (a) Education, (b) Health and Environment, and (c) Social and Economic. The Education domain consists of indicators such as early childhood education centers and Advanced Placement (AP) course enrollment, the Health and Environment domain consists of indicators such as access to greenspace and airborne microparticles concentration, and the Social and Economic domain consists of indicators such as median household income and number of single parent headed households. As expected, higher COI values are associated with better child health outcomes, such as reduced cortisol (an indicator of stress), asthma‐related hospitalizations, and pediatric acute care visits (Beck et al., 2017; Kersten et al., 2018; Krager et al., 2021; Roubinov et al., 2018) and higher frequency of psychiatric emergencies (Chen et al., 2022).

The primary aim of the present study is to examine whether poorer neighborhood resources and conditions, as measured by the COI at birth, are related to ADHD symptoms during mid‐childhood/adolescence. We will also investigate whether the effect of COI at birth differs in autistic individuals relative to those with DD (without autism) or TD individuals. The COI at birth is presumed to reflect the neighborhood conditions experienced during the latter part of gestation and a portion of early childhood. This period represents a crucial point of rapid brain development (Tierney & Nelson, 2009) and few life‐course studies have examined the effect of early life environmental exposures on outcomes throughout adolescence in autistic individuals. Data were leveraged from the CHARGE (Childhood Autism Risks from Genetics and the Environment) and ReCHARGE Study, which compared autistic youth to two comparison groups – those with developmental delays (DD) without autism and typically developing (TD) children. The inclusion of the DD group as a comparison group controls for confounding variables, such as developmental trajectories (e.g., language delays). All children were evaluated at ages 2–5 years (CHARGE) and subsequently assessed at ages 8–20 years (ReCHARGE).

In this study, we hypothesize that lower COI (poorer neighborhood conditions) at birth will be associated with greater ADHD symptoms in mid‐childhood/adolescence. While those with DD have been shown to have elevated ADHD symptoms relative to TD youth (Baker et al., 2010) no firm a priori hypothesis was made regarding interactions of COI with early childhood diagnostic status on ADHD symptoms, since we did not identify any prior study of this question. Poorer neighborhood conditions are associated with moderate risk for developmental disabilities (Blair & Ford, 2019) and greater symptoms of autism (Delobel‐Ayoub et al., 2015; Simonoff et al., 2020), but to our knowledge, the effect of environmental exposures on ADHD symptoms in those with autism and those with DD without autism have never been examined.

We will also explore which of the COI domains (Social and Economic, Health and Environment, Education) predicts ADHD symptoms. We hypothesize that Social and Economic and Education COI domain scores will predict ADHD symptoms given that educational and social economic characteristics of the neighborhood are consistently found to impact ADHD (Cheesman et al., 2022; Razani et al., 2015; Sundquist et al., 2015; Xue et al., 2005), while research regarding health and environmental characteristics, such as air pollution (Zhang et al., 2022) is mixed.

## METHODS

### Participants

CHARGE is an on‐going, large, population‐based case‐control study that has been enrolling children during early childhood—at ages 2‐5 years—since 2003. Details of the study have been described previously (Hertz‐Picciotto et al., 2006). Briefly, participants consisted of three groups – autistic children (AUT), children with developmental delay without autism (DD) and children with typical development (TD). The AUT and DD groups were recruited from regional centers and the TD group was recruited from population‐based data sources. The AUT group met cut‐off scores on the Autism Diagnostic Observation Schedule, Second Edition (ADOS‐2) and Autism Diagnostic Interview‐Revised (ADI‐R). The DD group had scores <70 on either the Mullen Scales of Early Learning (MSEL) or the Vineland Adaptive Behavior Scales (VABS), with the other score ≤76. Controls were recruited from birth records from a random sample of the general population, and assigned to the TD group if they scored ≥70 on the MSEL and the VABS. Individuals from the TD and DD group were screened for autism using the Communication Questionnaire (SCQ). Of those with SCQ scores that exceeded the clinical cut‐off (SCQ ≥15) or who were suspected of having an autism diagnosis based on a clinician's judgment, were further evaluated using the ADOS‐2 and ADI‐R to rule out an autism diagnosis. Of note, the TD group was recruited to match the sex distribution of the AUT group.

Participants were reconsented for follow‐up during mid‐childhood/adolescence at age 8–20 years as part of the ReCHARGE Study. Inclusion criteria for the present study includes (a) meeting the CHARGE Study criteria for any of the three diagnostic groups (AUT, DD, TD); (b) California residency at birth and (c) no sensory or physical impairments that would interfere with ability to complete the assessment.

### Ethical considerations

Informed consent has been appropriately obtained during participation at both CHARGE and ReCHARGE. The Institutional Review Board approved the project.

### Measures

#### Child opportunity index 2.0

Neighborhood conditions were measured using Child Opportunity Index 2.0 (COI; diversitydatakids.org) domain and overall scores. The COI dataset is publicly available and includes information for nearly all U.S. census tracts. The COI is based on 29 indicators across 3 domains: Education, Health and Environment, Social and Economic. COI developers calculated domain scores as weighted averages of the standardized indicators (z‐scores) within each domain. Indicators are weighted to reflect the strength of association between selected adult health outcomes and economic outcomes (see Noelke et al., 2020 for details). Overall COI scores were calculated by averaging all three COI domain scores using a similar weighting approach. Each U.S. census tract was ranked on COI domain and overall scores, resulting in scores ranging from 1 (lowest opportunity) to 100 (highest opportunity).

In the secondary analyses of this study, our team used COI categorical levels, which divided census tracts into quintiles (labeled as very low‐opportunity, low‐opportunity, moderate‐opportunity, high‐opportunity, and very high‐opportunity neighborhoods), each containing 20% of the child population. Only the AUT group was included in this analysis. Our team then constructed High and Low groups within the Social and Economic domain, with the Low‐Opportunity group (*n* = 93) consisting of the two lowest levels (very low‐ and low‐opportunity neighborhoods) and the High‐Opportunity group (*n* = 99) consisting of the two highest levels (high‐opportunity, and very high‐opportunity neighborhoods). Participants from the AUT group who were in the moderate‐opportunity range (*n* = 54) did not fall into either the Low or High Opportunity group and were therefore excluded from the secondary analyses.

To obtain the corresponding census tracts for each participants' address, geocoding was performed using ArcMap. Participants' 2010 census tracts were then merged with the publicly‐available COI dataset using SAS. Participant's addresses at the time of the child's birth were reported by the mother, which was compared and validated with the maternal addresses reported in the child's birth records. If mother's report and child's birth record did not match (*n* = 28), mother's report was used.

#### ADHD symptoms

The outcome variable was measured using The Aberrant Behavior Checklist (ABC) (Aman et al., 1985), which was administered at mid‐childhood/adolescence during their participation in ReCHARGE. The ABC is a 58‐item informant‐rated measure designed to assess problem behaviors in children. Each item is scored from 0 (not at all a problem) to 3 (problem in severe degree) across five subscales: Irritability (15 items), Lethargy/Social Withdrawal (16 items), Stereotypic Behavior (7 items), Hyperactivity (16 items), and Inappropriate Speech (4 items) The ABC was used based on its utility in evaluating ADHD symptoms and adequate psychometric properties among very young children and youth with neurodevelopmental disabilities (Karabekiroglu & Aman, 2009; Norris et al., 2019; Rojahn et al., 2013) and was designed specifically for youth with developmental disabilities and autism spectrum disorders (Aman & Singh, 2017). We used Subscale IV, labeled as “Hyperactivity” in the ABC, as the primary outcome measure, which we refer to as “ADHD Symptoms” throughout the paper.

The ABC was also administered during early childhood at the initial assessment in CHARGE and was used in the secondary analyses.

#### Covariates

Child age at ReCHARGE enrollment, sex assigned at birth, race, ethnicity (Hispanic vs. non‐Hispanic), and gestational age at delivery served as covariates in statistical analysis because of their potential relevance to ADHD symptoms and/or developmental outcomes (Bozinovic et al., 2021). Additional covariates relating to family social economic status included (maximum household educational level) and presence of perceived financial hardship (yes/no) in the child's early life. Families were asked “was there a time between 3 months before pregnancy to the present when it was hard for you to pay for basic needs like food, housing, medical care and heating?” These SES variables were chosen based on their lower correlation with other SES variables (i.e., home ownership, insurance status) to avoid multicollinearity issues. There were 40 participants with missing household education categorical data at birth so educational level at time of CHARGE enrollment were used.

### Statistical analysis

All analyses were conducted using SPSS 28 (SPSS Inc., Chicago, USA). Group differences in demographic and clinical characteristics and COI were assessed using chi‐square for categorical variables (sex, race/ethnicity, educational level, financial hardship) and one‐way ANOVA with post‐hoc multiple comparison correction (Tukey's HSD) for continuous variables (age, gestational age, ABC scores, COI scores).

To estimate the association of COI at birth with ADHD symptoms during mid‐childhood/adolescence, we conducted general linear modeling using the PROCESS script for SPSS (Hayes, 2014). Due to violation of normality, ADHD symptoms, the primary outcome variable, was transformed using Box‐Cox transformation procedures and COI values were centered to minimize multicollinearity. To evaluate whether the relation between COI and ADHD symptoms differed depending on diagnostic group (AUT,TD, DD), COI served as the predictor and the multinomial diagnosis was evaluated as a moderator (also known as an effect modifier) with the AUT serving as the reference group. To further explore which aspect of the neighborhood was influencing ADHD symptoms, we ran three additional linear regression models with the three COI domains (Education, Health and Environment, Social and Economic) as predictors. All variables had more than 95% available data.

Based on these results, a secondary analysis was conducted to examine how the AUT group with Low‐ versus High‐Opportunity scores on the Social and Economic COI domain differs in their course of ADHD symptoms beginning from early childhood to mid‐childhood/adolescence using a mixed model 2 (Time: Early Childhood vs. Mid‐Childhood/Adolescence) x 2 (Social and Economic COI level: Low vs. High) ANOVA. Bonferroni corrected pairwise comparisons were also conducted.

## RESULTS

### Participant characteristics

In these analyses, there were 246 autistic (AUT) children, 85 children with DD, and 193 TD controls. The mean age was 3.8 (SD = 0.79) years at the early childhood assessment for CHARGE and 13.48 (SD = 3.69) years at their participation in ReCHARGE; the mean gestational age was 38.84 (SD = 2.61) weeks; 76.5% children were male, 48% non‐White, and 28.3% Hispanic. Seventeen percent of families reported experiencing financial hardship at the time of child's birth. The maximum education level in the household at the time of child's birth was reported as 7.0% with a high school diploma or less, 27.8% with some college, 38.9% with a bachelor's degree and 26.3% with a graduate degree. Table 1 presents summary demographic and clinical characteristics for the three groups.

**TABLE 1:** Demographic and clinical characteristics of participants.

	AUT (*n* = 246)	DD (*n* = 85)	TD (*n* = 193)	Total (*n* = 524)	*p*‐value	Group differences
Age at CHARGE (years), mean (*SD*)	3.87 (0.8)	3.83 (0.73)	3.68 (0.8)	3.8 (0.79)	0.04	TD < AUT
Age at ReCHARGE (years), mean (*SD*)	13.68 (3.90)	12.77(3.65)	13.42(3.46)	13.48(3.69)	0.47	
Year of birth, mean (*SD*)	2005 (4.04)	2006 (3.75)	2005 (3.51)	2005.82 (3.81)	0.34	
Gestational age (weeks), mean (*SD*)	39 (2.32)	37.45 (3.68)	39.22 (2.21)	38.84 (2.61)	<0.001	DD < TD, AUT
Sex^a^, *n* (%)					0.001^a^	DD < TD, AUT
Male	199 (80.9%)	52 (61.2%)	150 (77.7%)	401 (76.5%)		
Female	47 (19.1%)	33 (38.8%)	43 (22.3%)	123 (23.5%)		
Ethnicity, *n* (%)					0.42	
Non‐hispanic	176 (71%)	145 (75%)	56 (66%)	377 (72%)		
Hispanic	68 (28%)	48 (25%)	27 (32%)	143 (27%)		
Race, *n* (%)					0.05	
White	127 (51.8%)	40 (48.2%)	4 (53.9%)	271 (52%)		
Black	11 (4.5%)	6 (7.2%)	2 (1%)	19 (3.6%)		
Asian	18 (7.3%)	2 (2.4%)	7 (3.6%)	27 (5.2%)		
Multi‐racial	89 (36.3%)	35 (42.2%)	80 (41.5%)	204 (39.2%)		
MSEL early learning composite, mean (*SD*)	64.13 (22.97)	56.55 (16.42)	108.11 (13.44)	79.13 (29.25)	<0.001	DD < AUT < TD
Educational level *n* (%)						
High school Diploma/GED or less	19 (7.7%)	9 (4.7%)	14 (8.6%)	42 (7.0%)	0.27	
Some college	70 (28.5%)	45 (23.3%)	52 (32.1%)	167 (27.8%)		
Bachelor degree	95 (38.6%)	80 (41.5%)	59 (36.4%)	234 (38.9%)		
Graduate/Professional degree	62 (25.2%)	59 (30.6%)	37 (22.8%)	158 (26.3%)		
Financial hardship					0.03	
No	191 (80.3%)	169 (88.5%)	123 (78.8%)	483 (82.6%)		TD < AUT, DD
Yes	47 (19.7%)	22 (11.5%)	33 (21.2%)	102 (17.4%)		
ABC at CHARGE, mean (*SD*)						
Subscale IV: Hyperactivity	18.67 (10.78)	9.36 (8.59)	3.42 (5.49)	11.18(11.00)	<0.001	TD < DD < AUT
Hyperactivity/Impulsivity	11.32 (7.63)	5.57 (5.98)	2.11 (3.71)	6.97 (7.48)	<0.001	TD < DD < AUT
Inattention	4 (2.14)	1.61 (1.64)	0.59(1.07)	2.36 (2.35)	<0.001	TD < DD < AUT
Oppositionality/defiance	3.26 (2.2)	1.6 (1.79)	0.73 (1.17)	2.07 (2.16)	<0.001	TD < DD < AUT
ABC at ReCHARGE, mean (*SD*)						
Subscale IV: Hyperactivity	13.35 (11.00)	7.53 (8.89)	2.84 (6.10)	8.40 (10.13)	<0.001	TD < DD < AUT
Hyperactivity/Impulsivity	8.49 (7.73)	5.27 (6.26)	5.27 (6.26)	5.41 (7.06)	<0.001	TD < DD < AUT
Inattention	3.07 (2.21)	2.03 (1.9)	2.03 (1.9)	2.05 (2.17)	<0.001	TD < DD < AUT
Oppositionality/defiance	1.77 (2.05)	1.77 (2.2)	0.49 (1.2)	1.3 (1.92)	<0.001	TD < DD, AUT
Child opportunity index, mean (*SD*)						
Social and economic	52.29 (26.51)	49.73 (27.54)	56.07 (25.00)	53.28 (26.18)	0.38	
Health and environment	62.96 (23.98)	58.86 (21.27)	63.46 (23.10)	62.48 (23.25)	0.29	
Education	52.82 (31.79)	47.44 (29.85)	51.91 (30.08)	51.61 (30.85)	0.13	
Overall COI	54.7 (27.32)	50.28 (27.01)	56.95 (25.58)	54.82 (26.68)	0.16	

Abbreviations: COI, Child Opportunity Index 2.0; MSEL, Mullen Scales of Early Learning; One‐way ANOVA with post‐hoc multiple comparison correction (Tukey's HSD).

^a^ TD group was recruited to match gender distribution of AUT group.

Post‐hoc comparisons indicated TD children were younger (*M* = 3.68 years, *SD* = 0.8) than autistic children (*M* = 3.87 years, *SD* = 0.8) at CHARGE enrollment (*p* = 0.035). Relative to the other two groups, the DD group had a higher ratio of females to males (*p* < 0.001), a lower gestational age (*p* < 0.001) and by definition, a lower MSEL Early Learning Composite (*p* < 0.001). The TD group reported lower rates of financial hardship (*p* < 0.05). The three groups were similar in all other demographic and SES variables (*p* > 0.05). There were also no group differences in neighborhood opportunity scores; the mean Child Opportunity Index ranged from 47.44 to 63.46 across all domain (Social and Economic, Health and Environment, Education) and overall scores.

As expected, the AUT group had significantly greater ADHD symptoms compared to each of the other groups. Similarly, the DD participants had greater symptoms compared with TD (*p* < 0.001 from *t*‐tests for all comparisons).

### Association between COI and ADHD symptoms

We examined correlations between all neighborhood opportunity scores and ADHD symptoms at mid‐childhood/adolescence (Table 2). Across the entire sample, there were small, but significant negative correlations between overall COI and ADHD symptoms (*r* = −0.115, *p* < 0.01). Together, this indicates the fewer the neighborhood opportunities and resources experienced in early childhood, the more ADHD symptoms youth exhibit in mid‐childhood/adolescence.

**TABLE 2:** Correlations between neighborhood opportunities and ADHD symptoms.

	Overall COI	COI social and economic	COI education	COI health and environment	ReCHARGE ABC subscale IV
Overall COI	–				
COI social and economic	0.962**	–			
COI education	0.873**	0.729**	–		
COI health and environment	0.671**	0.573**	0.550**	–	
ReCHARGE ABC (ADHD) subscale IV	−0.115**	−0.115**	−0.093*	−0.058	–
ReCHARGE ABC hyperactivity	−0.106**	−0.105**	−0.087*	−0.059	0.973**
ReCHARGE ABC inattention	−0.122**	−0.121**	−0.098*	−0.054	0.868**

*Abbreviations*: COI, Child Opportunity Index 2.0; ABC, Aberrant Behavior Checklist.

* Correlation is significant at the 0.05 level (2‐tailed).

** Correlation is significant at the 0.01 level (2‐tailed).

The COI domain scores of Social and Economic (*r* = −0.115, *p* < 0.01) and of Education (*r* = −0.093, *p* < 0.01) correlated negatively with ADHD symptoms. There were no significant correlations between Health and Environment COI domain scores and ADHD symptoms. See Table 2 for details.

### Early childhood predictors of ADHD in mid‐childhood/adolescence

Regression analyses were conducted to assess whether poorer neighborhood resources and conditions, as measured by the COI at birth, are related to ADHD symptoms during mid‐childhood/adolescence and whether this relation was moderated by diagnostic group. Race/ethnicity and SES variables (family hardship, household education) did not add significant variance above and beyond COI and were thus removed from the model due to their significant correlation with COI. The model with race/ethnicity and SES predictors can be found in Supplemental Table 1. We also ran the analyses and included early childhood (CHARGE) ADHD symptoms as a sensitivity analyses, and found it did not affect the results in a meaningful way.

**FIGURE 1:**
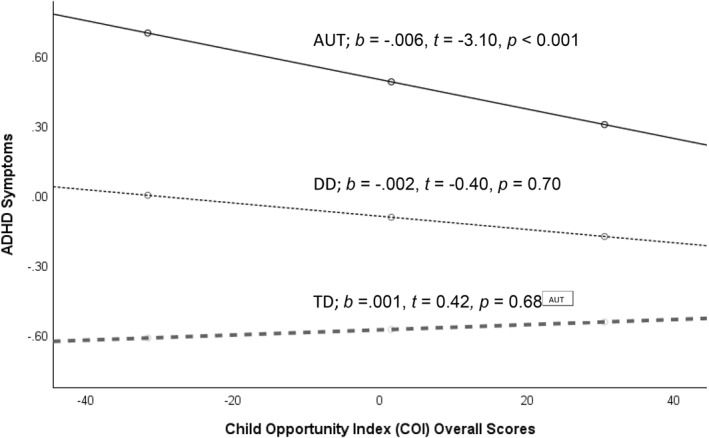
Simple Slopes of Neighborhood Opportunity on attention‐deficit/hyperactivity disorder (ADHD) Symptoms during Mid‐Childhood/Adolescence by Diagnostic Group. *Note*. Nationally normed overall Child Opportunity Index 2.0 (COI) scores are negatively related to ADHD symptoms in the AUT (autistic in solid line) group, whereas the relation between COI and ADHD symptoms is not significant in the DD (Developmental Delay in dotted line) and TD (Typically Developing in dashed line) groups. ADHD symptoms are log transformed ABC Subscale IV scores. Covariates in the model include age at mid‐childhood/adolescence, sex assigned at birth, and gestation age.

The final overall model was statistically significant, *R* = 0.53, F(8, 506) = 24.71, *p* < 0.001 (see Table 3). Age significantly predicted ADHD symptoms, indicating that the younger the participants were at their ReCHARGE assessment, the greater their ADHD symptoms. Sex assigned at birth and gestational age were not significantly related to ADHD symptoms. After adjusting for covariates, the COI was strongly associated with ADHD symptoms, indicating that the fewer the resources in the neighborhood, the greater the ADHD symptoms in mid‐childhood/adolescence. Relative to the AUT group, both the TD and DD groups had significantly fewer ADHD symptoms. There was also a significant interaction between the COI and early childhood diagnosis: AUT versus TD (*b* = 0.007*, t* = 2.28*, p =* 0.02). The interaction between COI and AUT versus DD was not significant. Post‐hoc analyses of simple slopes indicated that within the AUT group, COI significantly predicted ADHD symptoms, *b* = −0.006, *t* = −3.10, *p* < 0.001. In contrast, COI did not significantly predict ADHD symptoms within the TD or DD groups. See Figure 1.

To determine which of the COI domains predicted ADHD symptoms, three exploratory analyses were conducted with each of the COI domain scores (Social and Economic, Health and Environment, Education) as the predictor variable and early childhood diagnosis as the moderator/effect modifier. After accounting for covariates, the Social and Economic COI (*b* = −0.007, *t* = −2.99, *p* = 0.003) and the Education COI (*b* = −0.005, *t* = −2.94, *p* = 0.003) domains significantly predicted ADHD symptoms, whereas the Health and Environmental COI domain did not, though the slope was similarly negative. Similar to the results for the overall COI, significant interactions were also observed comparing AUT versus TD (*b* = −0.008, *t* = 2.21, *p* = 0.03) for the Social and Economic COI domain, but not for the other two domains. Post‐hoc analyses of simple slopes indicated that within the AUT group, the Social and Economic COI domain significantly predicted ADHD symptoms, *b* = −0.007, *t* = −2.99, *p* = 0.003. In contrast, the Social and Economic COI did not significantly predict ADHD symptoms within the TD or DD groups. No other interaction was significant in relation to ADHD symptoms (see Supplemental Table 2). This finding indicates that the social factors and economic resources of the neighborhood may be particularly influential in predicting which autistic youth are at risk for ADHD symptoms in mid‐childhood/adolescence.

**TABLE 3:** Predictors of ADHD symptoms during mid‐childhood/adolescence.

	B	t	*p*	*R* ^2^
Full model			<0.001	0.28
Overall COI	−0.006	−3.048	0.002	
AUT	Ref			
DD	−0.459	−3.894	<0.001	
TD	−1.075	−12.663	<0.001	
AUT * overall COI	Ref			
DD * overall COI	0.008	1.847	0.065	
TD * overall COI	0.007	2.276	0.023	
Sex^a^	−0.032	−0.342	0.732	
Gestational age	0.014	0.909	0.364	
Age at ReCHARGE	−0.005	−5.380	<0.001	

*Note*: COI, Child Opportunity Index 2.0; Regression analyses revealed neighborhood factors, as measured by the Childhood Opportunity Index (COI) at birth, significantly predicted ADHD symptoms during mid‐childhood/adolescence and that early childhood diagnosis modified this relation. The Autism (AUT) group served as the reference group for individuals with developmental delays (DD) and those that are typically developing (TD).

^a^ Males were used as reference group.

### Change in attention‐deficit/hyperactivity disorder symptoms from early childhood to mid‐childhood/adolescence in autistic youth: High versus. low social and economic opportunities

**FIGURE 2:**
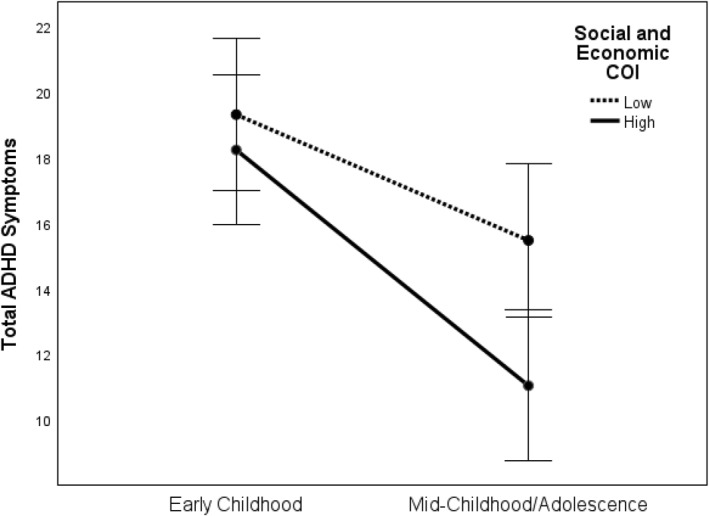
Change in attention‐deficit/hyperactivity disorder (ADHD) Symptoms Across Time in Autistic Youth: Low versus High Social and Economic Opportunities. *Note*. Both autistic groups have similar levels of ADHD symptoms, as measured by the Aberrant Behavior Checklist (ABC) Subscale IV, during early childhood (age 2–5 years), but those in the Low Social Economic Child Opportunity Index 2.0 (COI) group have significantly higher ADHD symptoms during mid‐childhood/adolescence (age 8–20 years), after controlling for age, sex, and gestational age. Error bars: 95% CI.

Based on the significant interaction between an early childhood diagnosis and Social and Economic COI described previously, an additional mixed model ANOVA was conducted to assess how autistic youth with low and high Social and Economic COI domain scores differ in their time course of Total ADHD Symptoms from early childhood to mid‐childhood/adolescence). Results revealed a significant main effect of Time, *F* (1,173) = 27.81, *p* < 0.001, no main effect of Social and Economic COI domain scores (*p* > 0.05), and no interaction (*p* > 0.05). Bonferroni corrected pairwise comparisons indicated both the low and high Social and Economic COI domain score groups had similar levels of ADHD symptoms during early childhood (*p* = 0.51), but by mid‐childhood/adolescence, autistic youth with low Social and Economic COI scores had significantly higher ADHD symptoms than those with higher Social and Economic COI scores (*p* < 0.01). See Figure 2.

## DISCUSSION

This is the first published study to our knowledge to examine whether poorer neighborhood resources and conditions at birth are related to ADHD symptoms during mid‐ childhood/adolescence in autistic youth or those with developmental delays without autism. Consistent with our hypothesis, poorer neighborhood opportunities were associated with ADHD symptoms. However, early childhood diagnosis moderated this association, such that the association between COI and ADHD symptoms was only present in the AUT group and not in the DD or TD groups.

While associations between neighborhood environments and ADHD prevalence have been reported elsewhere (Butler et al., 2012; Razani et al., 2015), it was unexpected that neighborhood exposures would have a greater effect on autistic individuals relative to those with DD without autism and those that are TD. These findings suggest that autistic persons may be particularly vulnerable to neighborhood environmental factors during gestation and early childhood, given their unique neurodevelopmental differences relative to those with those with DD without autism and those that are TD.

There is evidence that neighborhood disadvantages are associated with aberrant neural functioning in brain regions that are also affected in autistic individuals. For example, neighborhood disadvantage is associated with decreased prefrontal and hippocampal volume (Taylor et al., 2020) as well as alterations in functional connectivity across higher‐order (e.g., default mode network and dorsal attention network) and sensorimotor functional systems (Rakesh, Zalesky, & Whittle, 2021). These neural differences in turn are associated with poorer executive functioning and emotional and behavioral problems (Rakesh, Seguin, et al., 2021) that are prominent in autism. While those with DD without autism also exhibit neurocognitive and behavioral differences, ADHD symptoms in this group may be less malleable by environmental factors, or potentially, they may be masked by a low level of cognitive functioning. In fact, most of the correlation between ADHD and intellectual disability liabilities has been shown to be explained by genetic factors (estimated at 91%; Faraone et al., 2017). It is also likely that there are fewer resources for addressing ADHD in poorer neighborhoods. In line with the idea that autistic individuals may be more vulnerable to environmental risks than those with those with DD without autism and their TD peers, there is evidence that poorer environmental conditions (e.g., low neighborhood SES) are related to poorer language development (Grandgeorge et al., 2009), more emotional problems (Flouri et al., 2015) and poorer health behaviors (Fiscella et al., 2021) in autistic persons. Additionally, other researchers have reported greater neighborhood deprivation significantly predicts a higher relative increase in autism symptoms over time (Delobel‐Ayoub et al., 2015; Simonoff et al., 2020).

When examining the three COI domains separately, the interaction between early childhood diagnostic status and COI was only observed for the Social and Economic domain of the COI, suggesting that it is the social and economic characteristics of the neighborhood that have more of an effect on ADHD symptoms for autistic children than for TD or DD children. This is somewhat expected given that most neighborhood research mentioned above employ neighborhood SES as a proxy for overall neighborhood resources. However, in addition to using indicators of neighborhood wealth (i.e., poverty rate, public assistance rate, high‐skill employment, median household income and homeownership rate), our study's use of the COI also covers other characteristics, such as access to jobs (i.e., percentage of adults who are employed and spatial proximity to employment) and rates of single‐headed households. Neighborhoods high in these measures may have more financial resources to invest in amenities (e.g., schools, parks, after‐school programs) and attract more private business and service providers, and as such, it is no surprise that children with greater neighborhood economic resources would, on average, experience more positive outcomes. Notably, in our study, we found no difference in ADHD symptoms at ages 2–5 years comparing autistic youth with high versus low social and economic neighborhood opportunities, but by mid‐childhood/adolescence, autistic youth with high social and economic neighborhood opportunities have significantly lower ADHD symptoms. While both groups of autistic youth experience elevated ADHD symptoms relative to their TD peers, it is likely that greater access to economic and social resources facilitated improvement of ADHD symptoms. This hypothesis of the current study, that neighborhood, above and beyond family factors, is a critical influence on neurodevelopment such as ADHD, is possibly corroborated by evidence that children living in relatively affluent neighborhoods improve in ADHD symptoms over time, regardless of familial risk factors such as household SES or family conflict (Sharp et al., 2021).

The current findings may provide insight into policy efforts, as it highlights the importance of increased and improved resources for children with neurodevelopmental concerns living in poor neighborhoods. Despite the well‐established efficacy of interventions for autism and ADHD (Makrygianni et al., 2018; Vismara & Rogers, 2010), children living in poorer neighborhoods have less access to related services (Cantor et al., 2022), likely contributing to racial, ethnic and sociodemographic disparities in both identification of autism and ADHD (Aylward et al., 2021; Coker et al., 2016; Singh & Bunyak, 2019) and treatment utilization (Smith et al., 2020; Zuckerman et al., 2017). The identification and treatment of ADHD during childhood has the potential to change an individual's life trajectory. ADHD is associated with significant adverse academic and social outcomes during childhood, which continue to lead to adverse outcomes later in life, such as higher suicidality, greater likelihood of incarceration, lower college matriculation and graduation rates, and lower levels of occupational attainment and employment (Arnold et al., 2020; Hechtman et al., 2016; Shoham et al., 2021). ADHD treatment (i.e., medications and/or behavioral therapy) for those with and without autism is critical to improving these long‐term outcomes. As such, federal and state agencies, private organizations, and academic institutions are encouraged to support research, services, and policy initiatives to address inequities within the neurodevelopmental disability community. More specifically, the present findings highlight the need for the assessment of ADHD symptoms within the autistic population and the development of low cost, easily disseminated programs and policies to address ADHD symptoms specifically for autistic youth. A promising example is the relatively recent Executive Order On Advancing Racial Equity and Support for Underserved Communities Through the Federal Government which has resulted in equity activities across every federal agency. Another potential strategy to address inequities is providing parent training workshops at community primary care clinics or a family navigation program, which involves a trained navigator who guides families through and around barriers to ensure timely diagnosis and treatment (Freeman, 2012).

### Strengths and limitations

Multiple strengths of the present study should be noted: a relatively diverse sample that includes strong representation of Hispanics and multi‐racial participants; application of rigorous diagnostic criteria both for early childhood neurodevelopmental conditions and for ADHD in mid‐childhood through adolescence; adjustment for a range of potential confounders; a validated, nationally‐normed census tract level measure of neighborhood conditions; and longitudinal data to characterize changes from early childhood through adolescence. Furthermore, in addition to the TD group, we included a second comparison group of those with DD without autism. In contrast to the significant focus and resources directed toward autism research, there is notably less attention given to individuals experiencing DD without autism. Interestingly, although group differences in COI did not reach significance, it is noteworthy that the DD group exhibited the lowest COI values compared to the AUT and TD groups. This underscores a broader social justice issue, suggesting DD youth may be exhibiting more pronounced disparities. Thus, by incorporating this crucial demographic, we are enhancing the representation of diverse populations in the neurodevelopmental literature.

Nevertheless, several limitations warrant consideration. The ABC was used to assess ADHD symptoms based on its adequate psychometric properties among very young children and youth with neurodevelopmental disabilities (Karabekiroglu & Aman, 2009; Norris et al., 2019; Rojahn et al., 2013), however, ratings are based on a single informer (parent or guardian) which may be confounded by biases. Also, while the use of the COI allowed for measurement of several relevant neighborhood indicators, the COI does not include other factors that may influence child outcomes, such as perceived safety and social support (Dahal et al., 2018) or vandalism (Butler et al., 2012). In addition, the COI at birth provides a proxy for the neighborhood conditions experienced during the latter part of gestation and some portion of early childhood, but we did not account for the possibility of families moving during pregnancy or early childhood. This includes families that lack stable housing, transition between neighborhoods, split households (i.e., divorced parents), or those that shift between transient and stable states of poverty, and future research should take these situations into consideration. Similarly, while the sample was relatively large and diverse (about 28% identify as Hispanic), the sample was still relatively higher in SES compared to the general population, as evidenced by the high education levels (>50% had bachelor's degree or higher) and low rates of financial hardship (17.5%). This may explain why there was no significant relation found between the COI and ADHD symptoms in the TD or DD groups. It may be possible that COI influences ADHD symptoms among TD and DD individuals from much lower SES communities, but we lacked sufficient power to detect it. However, the presence of a significant effect of neighborhood factors on ADHD symptoms in the AUT group, despite the relatively high SES of the sample, indicates how influential neighborhood conditions are for those with autism. Nonetheless, more robust and inclusive research is encouraged, which might necessitate adept strategies for engaging with individuals and communities from lower SES backgrounds such as recruiting directly from community settings or customizing study procedures to accommodate participants' situations (Emery et al., 2023). Another limitation is the wide age range (8–20 years) during the ReCHARGE assessment conducted during mid‐childhood/adolescence. Age at ReCHARGE significantly predicted ADHD symptoms, indicating that the older participants had fewer ADHD symptoms, consistent with the known tendency for ADHD symptoms to decline across time (O'Neill et al., 2017). We attempted to control for the age effect by including age at ReCHARGE as a covariate, however future research comparing the effect and relative importance of neighborhood conditions across childhood, adolescence and adulthood is warranted. Lastly, we did not assess or adjust for the effects of pharmacological or psychosocial treatment; however, it is highly likely that engagement in treatment is largely associated with neighborhood conditions given the known racial, socioeconomic and geographical disparities in treatment utilization (Angell et al., 2018; Yingling et al., 2019).

## CONCLUSION

We expand the literature indicating that the neighborhood in which a child lives has a significant impact on health and well‐being to include the course of a child's symptoms of ADHD. Our study shows that even after accounting for child demographics and family socioeconomic factors, neighborhood resources and conditions experienced during early childhood are related to ADHD symptoms in mid‐childhood/adolescence, particularly in autistic individuals. These findings have important clinical implications and emphasize the need for increased and improved resources in poorer neighborhoods to reduce existing disparities that have lifelong impacts.

## AUTHOR CONTRIBUTIONS

**Catrina A. Calub**: Conceptualization; Formal analysis; Methodology; Writing – original draft. **Irva Hertz‐Picciotto**: Data curation; Investigation; Project administration; Resources; Supervision; Writing – review & editing. **Deborah Bennett**: Conceptualization; Funding acquisition; Investigation; Project administration; Resources; Supervision; Writing – review & editing. **Julie B Schweitzer**: Funding acquisition; Investigation; Project administration; Resources; Supervision; Writing – review & editing.

## CONFLICT OF INTEREST STATEMENT

The authors have declared no competing or potential conflicts of interest.

## ETHICS STATEMENT

Informed consent has been appropriately obtained during participation at both CHARGE and ReCHARGE. The UC Davis Institutional Review Board approved the project.

## Supporting information

Supplementary Material

## Data Availability

Data are not currently publicly available but can be available upon request from Dr. Catrina Calub at ccalub@ucdavis.edu.
