# Supplementary material for: Examining the association of neighborhood conditions on attention‐deficit/hyperactivity disorder symptoms in autistic youth using the child opportunity index 2.0
Source: JCPP Adv. 2024 Jul 18;5(1):e12267. doi: 10.1002/jcv2.12267 (PMC11889653; doi:10.1002/jcv2.12267)
Supplement: Supplementary file 1 — Supplementary Material [file JCV2-5-e12267-s001.docx]

| Table S1. | | | | |  |
| --- | --- | --- | --- | --- | --- |
| Predictors of ADHD Symptoms During Mid-Childhood/Adolescence | | | | | |
|  | **B** | **t** | **p** | **R^2^** | |
| Full Model |  |  | <0.001 | .28 | |
| Overall COI | -0.0063  0.0063 | -2.6064 | 0.0095 |  | |
| AUT | Ref |  |  |  | |
| DD | -0.3233 | -2.4656 | 0.0141 |  | |
| TD | -0.9985 | -10.538 | <0.001 |  | |
| AUT * Overall COI | Ref |  |  |  | |
| DD * Overall COI | 0.0069 | 1.5882 | 0.1131 |  | |
| TD * Overall COI | 0.0089 | 2.5116 | 0.0125 |  | |
| Sex^a^ | -0.101 | -0.9825 | 0.3265 |  | |
| Gestational Age | 0.0107 | 0.625 | 0.5324 |  | |
| Age at ReCHARGE | -0.0046 | -4.2016 | <0.001 |  | |
| Race^b^ | 0.005 | 0.3436 | 0.7314 |  | |
| Ethnicity^c^ | 0.1392 | 1.3935 | 0.1643 |  | |
| Education^d^ | -0.0127 | -0.2558 | 0.7982 |  | |
| Financial Hardship^e^ | -0.0986 | -0.5419 | 0.5882 |  | |
| Note. Regression analyses revealed neighborhood factors, as measured by the Childhood Opportunity Index 2.0 (COI) at birth significantly predicted ADHD symptoms during mid-childhood/adolescence and that early childhood diagnosis moderated this relation. The Autism (AUT) group served as the reference group for individuals with developmental delays (DD) and those that are typically developing (TD). ^a^Males used as reference group. **^b^**White used as reference group. ^c^Non-hispanic used as reference group. ^d^High School Dipoloma/GED or less used as reference group^. e^No financial hardship used as reference group. | | | | | |
|  |  |  |  |  |  |
|  | | | | |  |

| Table S2. |  |  |  |  |
| --- | --- | --- | --- | --- |
| Predictors of ADHD Symptoms During Adolescence | | | | |
|  | **B** | **t** | **p** | **R^2^** |
| Full Model | |  | <0.001 | 0.27 |
| COI Health and Environment | -0.003 | -1.2443 | 0.214 |  |
| AUT | Ref |  |  |  |
| DD | -0.458 | -3.8615 | 0.0001 |  |
| TD | -1.0728 | -12.582 | <0.001 |  |
| AUT * Overall | Ref |  |  |  |
| DD * COI Health and Environment | 0.0068 | 1.3021 | 0.1935 |  |
| TD * COI Health and Environment | 0.0042 | 1.1314 | 0.2584 |  |
| Sex | -0.0175 | -0.1865 | 0.8521 |  |
| Gestational Age | 0.0124 | 0.8096 | 0.4186 |  |
| Age | -0.0049 | -5.5637 | <0.001 |  |
| Full Model | |  | <0.001 | 0.28 |
| COI Education | -0.0053 | -2.9398 | 0.0034 |  |
| AUT | Ref |  |  |  |
| DD ^a^ | -0.4773 | -4.0663 | 0.0001 |  |
| TD ^a^ | -1.077 | -12.712 | <0.001 |  |
| AUT * COI Education | Ref |  |  |  |
| DD * COI Education | 0.0047 | 1.2454 | 0.2135 |  |
| TD * COI Education | 0.0052 | 1.8634 | 0.063 |  |
| Sex | -0.0314 | -0.3374 | 0.7359 |  |
| Gestational Age | 0.0128 | 0.845 | 0.3985 |  |
| Age | -0.0048 | -5.49 | <0.001 |  |
| Full Model | |  | <0.001 | 0.28 |
| COI Social and Economic | -0.0066 | -2.9909 | 0.225 |  |
| AUT | Ref |  |  |  |
| DD | -0.4689 | -3.986 | 0.0029 |  |
| TD | -1.0782 | -12.67  12.6707 | 0.0001 |  |
| AUT * COI Social and Economic | Ref |  |  |  |
| DD * COI Social and Economic | 0.0068 | 1.586 | <0.001 |  |
| TD * COI Social and Economic | 0.0076 | 2.2153 | 0.1134 |  |
| Sex ^b^ | -0.0307 | -0.3299 | 0.0272 |  |
| Gestational Age | 0.0139 | 0.913 | 0.7416 |  |
| Age | -0.0047 | -5.406 | 0.3617 |  |
| **Note.** COI = Child Opportunity Index 2.0. Regression models of early childhood predictors on ADHD symptoms during adolescence ^a^Autism (AUT) group as reference group for the typically developing (TD) and developmental delay (DD) groups. ^b^Males used as reference group. | | | | |

| Table S3 |  |  |  |  |  |
| --- | --- | --- | --- | --- | --- |
| 2 (Time: Early Childhood vs Mid-Childhood/Adolescence) x 2 (Social and Economic COI level: Low vs High) ANOVA | | | | | |
|  | **SS** | **df** | **MS** | **F** | ***p*** |
| **Within Groups** |  |  |  |  |  |
| Time | 2659.70 | 1 | 22659.70 | 27.81 | <.001 |
| Time * Social and Economic COI | 245.97 | 1 | 245.97 | 2.57 | .11 |
| **Between Groups** |  |  |  |  |  |
| Diagnostic Group | 663.884 | 1 | 663.884 | 4.629 | .033 |
| Note. COI = Child Opportunity Index 2.0. Only autistic children included in this analysis | | | | | |
